# Cisplatin contributes to programmed death-ligand 1 expression in bladder cancer through ERK1/2-AP-1 signaling pathway

**DOI:** 10.1042/BSR20190362

**Published:** 2019-09-06

**Authors:** Te-Fu Tsai, Ji-Fan Lin, Yi-Chia Lin, Kuang-Yu Chou, Hung-En Chen, Chao-Yen Ho, Po-Chun Chen, Thomas I-Sheng Hwang

**Affiliations:** 1 Division of Urology, Department of Surgery, Shin-Kong Wu Ho-Su Memorial Hospital, Taipei, Taiwan; 2 Division of Urology, School of Medicine, Fu-Jen Catholic University, New Taipei, Taiwan; 3 Central Laboratory, Shin-Kong Wu Ho-Su Memorial Hospital, Taipei, Taiwan; 4 Institute of Traditional Medicine, School of Medicine, National Yang-Ming University, Taipei, Taiwan; 5 Department of Biotechnology, College of Health Science, Asia University, Taichung, Taiwan; 6 Department of Medical Research, China Medical University Hospital, China Medical University, Taichung, Taiwan; 7 Department of Urology, Taipei Medical University, Taipei, Taiwan

**Keywords:** Bladder cancer, cisplatin, ERK, PD-L1

## Abstract

Bladder cancer (BC) is the second most common urologic malignancy and the ninth most common malignancy worldwide. Surgical resection is the mainstay of treatment for patients with early-stage disease, whereas therapeutic options are limited for patients with advanced-stage or residual BC. Programmed cell death ligand-1 (PD-L1) is an important target for immunotherapy. It is known that PD-L1 is overexpressed in BC; a clinical trial involving PD-L1 immune checkpoint inhibitors in advanced BC is ongoing. In the present study, we used Western blot and quantitative real-time PCR (qPCR) to define the expression level of PD-L1 after cisplatin treatment in BC-derived cell lines. The signal activation was also evaluated by Western blot in BC-derived cell lines. We found that chemotherapeutic drug cisplatin can induce PD-L1 but not PD-L2 expression in BC-derived cell lines. Furthermore, the expression level of PD-L1 was increased in a dose- and time-dependent manner after cisplatin treatment. The cisplatin-induced PD-L1 expression is mainly mediated by ERK1/2 but not Akt/mTOR signal pathway. Moreover, we found that cisplatin activates transcription factor activator protein-1 (AP-1) to regulate PD-L1 expression. The chemotherapy drug such as cisplatin may trigger resistance of BC through PD-L1 up-regulation. The present study suggests that PD-L1 antibody should be used concomitantly with chemotherapy in the setting of advanced and metastatic BC.

## Introduction

Bladder cancer (BC) is a commonly diagnosed urological malignancy with a very high recurrence rate. The standard treatment for managing BC is a complete transurethral resection of the bladder tumor (TURBT). Intravesical chemotherapy including mitomycin C, epirubicin, and doxorubicin or Bacillus Calmette-Guérin (BCG) for non-muscle-invasive BC is generally used as adjuvant therapy after complete endoscopic resection [1]. For muscle-invasive BC, combinations of methotrexate, vinblastine, doxorubicin, and cisplatin (M-VAC) are recommended as first-line chemotherapy [2]. Currently, there are no definitive recommendations for second-line therapy. Novel, effective therapeutic options are warranted in the treatment of BC.

Evasion of the immune system is a hallmark of cancer. Cancer cells can express many immune inhibitory signaling proteins that cause immune cell dysfunction and apoptosis [3]. One of these inhibitory molecules is programmed cell death ligand-1 (PD-L1), which binds to programmed cell death 1 (PD-1) expressed on T cells, B cells, dendritic cells and natural killer T cells, to suppress anti-cancer immunity. Anti-PD-L1 and anti-PD-1 antibodies have been used for the treatment of cancer, including BC, with promising outcomes [4]. There are many ongoing clinical trials investigating the utility of PD-L1 in the treatment of BC [5]. Levels of PD-L1 expression correlate with BC severity and outcome. Tumors expressing higher levels of PD-L1 are more likely to be considered high-grade, and higher PD-L1 expression in urothelial cancer is more likely to result in postoperative recurrence and poorer survival than urothelial cancer with lower PD-L1 expression [6]. PD-L1 tumor cell expression is also associated with increased resistance to BCG therapy, which is thought to be related to immune system suppression, since a fully functioning immune system is required for BCG efficacy [7]. Overall, cancers with the highest mutational burden, such as BC, seem to benefit the most from immune checkpoint blockade therapy, because of the greater T-cell-mediated antitumor immune response elicited by these cancers [8].

Avelumab, an immune checkpoint inhibitor of the PD-L1/PD-1 axis, has shown anti-tumor activity in patients with platinum-refractory metastatic urothelial carcinoma [9]. However, the mechanisms that underlie the effects of immune checkpoint inhibitors have not been discussed previously. Here, we show that PD-L1 expression in BC cells is up-regulated after cisplatin treatment and that this is mediated mainly by ERK1/2/ activator protein-1 (AP-1) signal transduction. Our finding could explain the anti-tumor activity of immune checkpoint inhibition on platinum-refractory metastatic BC.

## Experimental

### Materials

Anti-mouse or anti-rabbit IgG–conjugated horseradish peroxidase, rabbit polyclonal antibodies specific for p-mTOR (Ser^2448^; cat. no. 2971), p-Akt (Ser^473^; cat. no. 4058), p-ERK1/2 (Thr^202^/Tyr^204^; cat. no. 4370), p-p38 (Thr^180^/Tyr^182^; cat. no. 9216), p-JNK (Thr^183^/Tyr^185^; cat. no.4668), p-c-jun (Ser^63^; cat. no. 9261), Akt (cat. no. 9272), p38 (cat. no. 9212), c-jun (cat. no. 9165), mTOR (cat. no. 2983), PD-L1 (cat. no. 13684) were purchased from Cell Signaling Technology (Danvers, MA, U.S.A.). ERK1/2 (cat. no. sc-94), JNK (cat. no. sc-7345), and β-Actin (cat. no. sc-47778) were purchased from Santa Cruz Biotechnology (Santa Cruz, CA, U.S.A.). Cisplatin (cat. no. 232120), Akt inhibitor (cat. no. 124005), PD98059 (cat. no. 19-143), U0126 (cat. no. 19-147), SP600125 (cat. no. S5567), SB203580 (cat. no. S8307) and Tanshinone IIA (cat. no. T4952) and all other chemicals were obtained from Sigma–Aldrich (St. Louis, MO, U.S.A.).

### Cell culture

Human BC cell lines (5637 and T24) were obtained from Bioresource Collection and Research Center (BCRC; Hsinchu, Taiwan) and maintained at 37°C under 5% CO_2_. 5637 and T24 cells were cultured in RPMI-1640 medium. Culture media were supplemented with 10% FBS, 2 mM GlutaMAX-1, 100 units/ml penicillin and 100 μg/ml streptomycin. Cells were seeded on plastic plates (96-well or 6-well) or 10-cm dishes for cell viability assays and collecting RNA or protein samples.

### Western blot analysis

The cellular lysates were prepared and proteins were then resolved on SDS/PAGE and transferred to Immobilon polyvinyldifluoride (PVDF) membranes. The blots were blocked with 4% BSA for 1 h at room temperature and then probed with rabbit primary antibodies against human proteins (1:1000) for 1 h at room temperature. After three washes, the blots were subsequently incubated with a donkey anti-rabbit peroxidase–conjugated secondary antibody (1:1000) for 1 h at room temperature. After three washes with TBST, the blots were then detected with Amersham ECL Western Blotting Detection Reagents (GE Healthcare; Chicago, IL, U.S.A.) and photographed by using a ChemiDoc-It® Imaging Systems (UVP Inc., Upland, CA, U.S.A.). Quantification of Western blot results was performed using ImageJ software1.49v (National Institute of Health, U.S.A.). β-actin was used as internal control and densitometry values of each detected proteins were normalized to β-actin. Results are expressed as the mean ± standard deviation (SD) of three independent experiments.

### Quantitative real-time PCR

The quantitative real-time PCR (qPCR) analysis was performed using Taqman® one-step PCR Master Mix (Applied Biosystems, Foster City, CA, U.S.A.). A total of 100 ng of total cDNA was added per 25-µl reaction with sequence-specific primers and Taqman® probes. Sequences for all target gene primers and probes were purchased commercially from Applied Biosystems (CA, U.S.A.); β-actin was used as the internal control. Quantitative RT-PCR assays were carried out in triplicate on a StepOnePlus sequence detection system. The cycling conditions were 10 min of polymerase activation at 95°C followed by 40 cycles at 95°C for 15 s and at 60°C for 60 s. The threshold was set above the non-template control background and within the linear phase of target gene amplification, to calculate the cycle number at which the transcript was detected (denoted as C_T_).

### Immunofluorescence microscopy

Cells grown on glass coverslips were rinsed with PBS and fixed in 3.7% formaldehyde for 10 min at room temperature. Cells were washed three times with PBS and blocked with 4% BSA for 15 min. Cells were then incubated with the p-c-Jun and PD-L1 primary antibody (1:100) for 1 h at room temperature, washed again, and incubated with FITC–conjugated secondary antibody (1:100) for 1 h. Finally, cells were washed, mounted with DAPI containing solution, and photographed with a Nikon Ti2 microscopy System.

### Statistics

All experiments were performed at least three times, each time in triplicate. Statistical comparisons between two samples were analyzed by Student’s *t* test for statistical significance and expressed as the means ± standard deviation (S.D.). A *P*<0.05 was considered statistically significant. The data containing more than two groups were performed using one-way analysis of variance (ANOVA) with Bonferroni’s post-hoc test. The difference was considered to be significant if the *P-*value was <0.05.

## Results

### Cisplatin treatment contributes to PD-L1 expression in BC-derived cell lines

**Figure 1:**
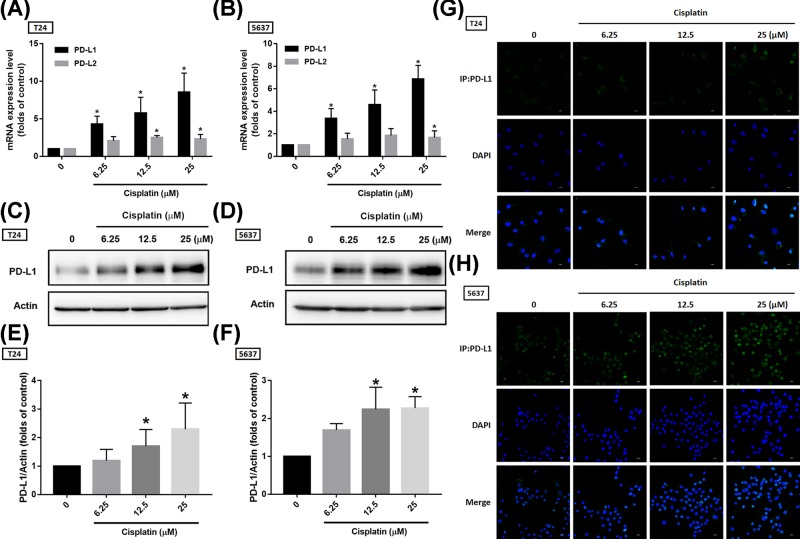
Cisplatin induces PD-L1 expression in a dose-dependent manner (**A,B**) T24 and 5637 BC-derived cell lines were treated with various concentrations of cisplatin for 24 h, total mRNA was extracted from cells, and expression levels of PD-L1 and PD-L2 were detected by qPCR. (**C,D**) T24 and 5637 BC-derived cell lines were treated with the indicated concentrations of cisplatin for 24 h, total protein was extracted and expression levels of PD-L1 were detected by Western blot. (**E,F**) The relative band intensities of proteins presented in (C,D) were quantified by densitometric scanning and are presented as the fold change of the control group. (**G,H**) The BC-derived cell lines were treated as (A,B) described, then the cells were performed with immunofluorescence staining by anti-PD-L1 antibody. Nuclei were counterstained with DAPI. Representative microscopy images are shown; the statistical calculation incorporates blots from three independent experiments. The results are presented as the mean ± S.D.; *P<0.05 compared with the control group.

**Figure 2:**
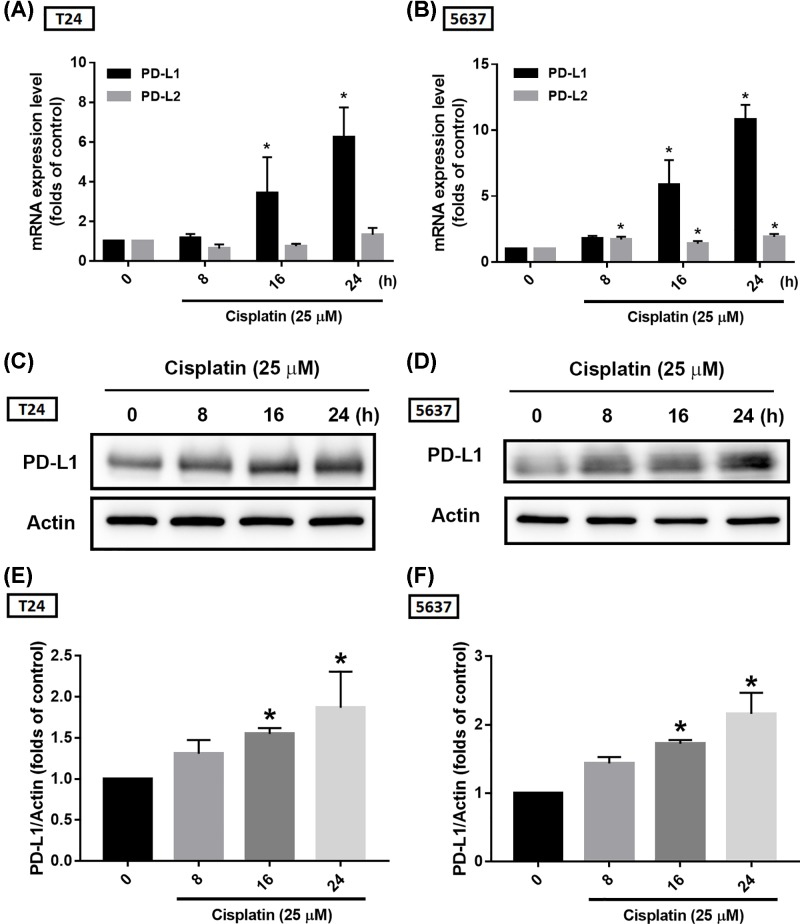
Cisplatin induces PD-L1 expression in a time-dependent manner (**A,B**) T24 and 5637 BC-derived cell lines were treated with 25 μM of cisplatin for 0, 8, 16 or 24 h, total mRNA was extracted from cells, and expression levels of PD-L1 and PD-L2 were detected by qPCR. (**C,D**) T24 and 5637 BC-derived cell lines were treated as described in (A,B), total protein was extracted and expression levels of PD-L1 were detected by Western blot. (**E,F**) The relative band intensities of proteins presented in (C,D) were quantified by densitometric scanning and are presented as the fold change of the control group; the statistical calculation incorporates blots from three independent experiments. The results are presented as the mean ± S.D.; *P<0.05 compared with the control group.

Since PD-1/PD-L1 expression is the main indication for these immune checkpoint inhibitors, and the expression of these immune checkpoint proteins is up-regulated with the progression of BC, it is reasonable to hypothesize that PD-L1 overexpression may be involved in the progression of BC by providing an escape route for tumor cells to evade immune detection. Suppression of these proteins by immune checkpoint inhibitors or other strategies may effectively treat BC. Our results found that cisplatin dose-dependently promoted PD-L1 mRNA expression but not that of PD-L2 (another ligand for PD-1), in BC-derived cell lines (Figure 1A,B). The protein expression was in accordance with mRNA expression (Figure 1C–F). We further confirmed PD-L1 expression via immunofluorescence staining and results also showed that cisplatin treatment improved PD-L1 expression in BC-derived cell lines (Figure 1G,H). Moreover, PD-L1 expression levels were increased after cisplatin treatment in a time-dependent manner (Figure 2). These findings show that cisplatin promotes PD-L1 expression in BC, suggesting chemoresistance via immune escape mechanisms.

### Cisplatin promotes PD-L1 expression in BC-derived cell lines mainly through ERK1/2 signal transduction

**Figure 3:**
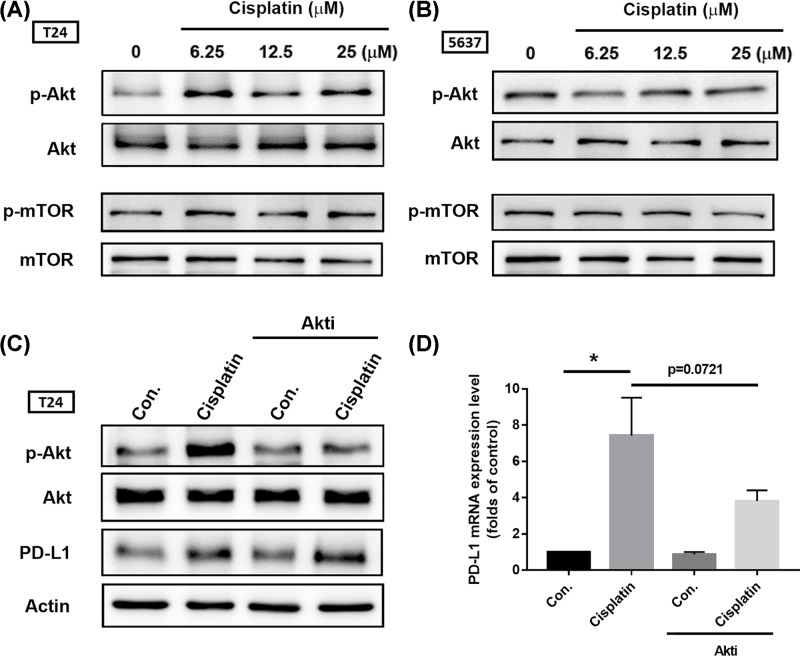
The Akt/mTOR signal pathway is not required for PD-L1 expression after cisplatin treatment (**A,B**) T24 and 5637 BC-derived cell lines were treated with different concentrations of cisplatin for 24 h (6.25, 12.5, or 25 μM, respectively), total protein was extracted and phosphorylation of Akt and mTOR was detected by Western blot. Total Akt and mTOR were used as the internal controls. (**C,D**) T24 and 5637 BC-derived cell lines were initially treated with 3 μM of Akti for 30 min, then with cisplatin (25 μM) for 24 h. Total protein was extracted and subjected to Western blot and qPCR assessments of Akt activation and levels of PD-L1. β-Actin was used as the internal control. Results are expressed as the mean ± S.D of triplicate samples. *P<0.05 compared with the control group.

**Figure 4:**
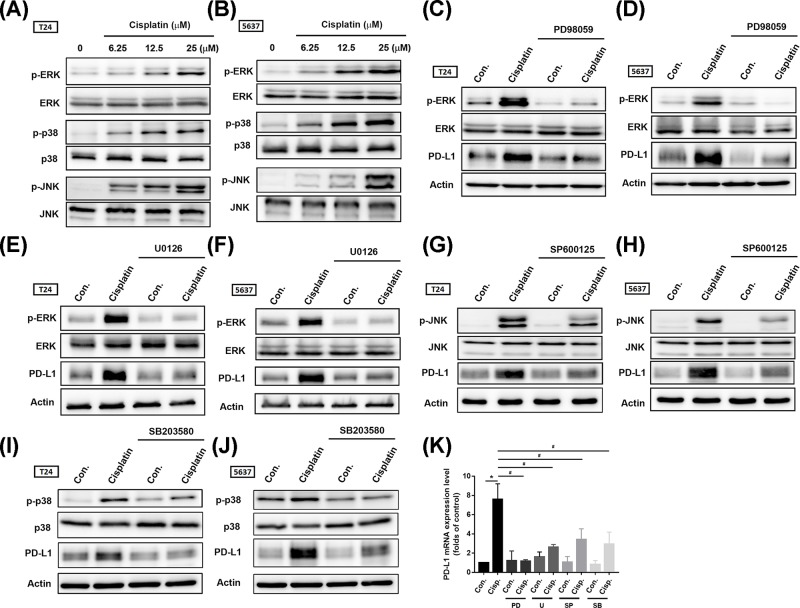
The ERK1/2 signaling pathway mainly contributes to PD-L1 expression after cisplatin treatment (**A,B**) T24 and 5637 BC-derived cell lines were treated with different concentrations of cisplatin for 24 h (6.25, 12.5, or 25 μM, respectively); total protein was extracted and phosphorylation of ERK1/2, p38 and JNK was detected by Western blot. Total ERK1/2, p38, and JNK were used as the internal controls. (**C–F**) T24 and 5637 BC-derived cell lines were pretreated with different ERK1/2 pathway inhibitors (PD98059, 10 μM; U0126, 10 μM) for 30 min then cisplatin (25 μM) for 24 h. Total protein was extracted, then ERK1/2 activation and expression levels of PD-L1 were detected by Western blot. β-Actin was used as the internal control. (**G,H**) T24 and 5637 BC-derived cell lines were pretreated with 10 μM of a JNK inhibitor (SP600125) for 30 min then with cisplatin (25 μM) for 24 h. Total protein was extracted and JNK activation and expression levels of PD-L1 were detected by Western blot. β-Actin was used as the internal control. (**I,J**) T24 and 5637 BC-derived cell lines were pretreated with 10 μM of p38 inhibitor (SB203580) for 30 min then with cisplatin (25 μM) for 24 h. Total protein was extracted, then p38 activation and expression levels of PD-L1 were detected by Western blot. β-Actin was used as the internal control. (**K**) T24 BC-derived cell lines were pretreated with different pathway inhibitors (PD = PD98059; U = U0126; SP = SP600125; SB = SB203580) for 30 min, followed by cisplatin (25 μM) incubation for 24 h, total mRNA was extracted from the cells and expression levels of PD-L1 were detected by qPCR. The efficacy of inhibitors was provided in Supplementary Figure S1. Results are expressed as the mean ± S.D of triplicate samples. *P<0.05 compared with the control group and #*P*<0.05 compared with the cisplatin-treated group.

Multiple mechanisms can contribute to intrinsic tumor PD-L1 expression. Previous research indicates that activation of the Akt/mTOR pathway promotes immune escape by driving PD-L1 expression in lung cancer [10]. Therefore, we first investigated Akt and mTOR activation after cisplatin treatment. We found that cisplatin promoted Akt phosphorylation rather than that of mTOR (Figure 3A,B); this effect was profound in T24 cells. Surprisingly, treatment with an Akt inhibitor (Akti) did not reverse cisplatin-induced PD-L1 expression in BC-derived cell lines (Figure 3C,D). We next screened for another candidate signal pathway by which cisplatin promotes PD-L1 expression. Earlier evidence has indicated that the mitogen-activated protein kinase kinase (MEK)/ERK signaling pathways play a critical role in the constitutive up-regulation of PD-L1 in cisplatin-resistant cells [11]. Mitogen-activated protein kinases (MAPKs) consist of a family of ubiquitous serine/threonine kinases that participate in signal transduction of extracellular hormones, growth factors and cytokines, which play a crucial role in immune responses [12]. MAPK signal cascade components were then evaluated after cisplatin treatment in BC-derived cell lines. Phosphorylation of MAPK signal components revealed the activation of ERK1/2, p38, and JNK after cisplatin treatment (Figure 4A,B). Moreover, pretreatment with ERK1/2 pathway inhibitors PD98059 and U0126 clearly inhibited ERK1/2 activation as well as PD-L1 expression after cisplastin incubation (Figure 4C–F). PD-L1 mRNA expression confirmed the involvement of ERK1/2 signal transduction (Figure 4K). In contrast, suppression of p38 and JNK activity had only a minor, but statistically significant effect on PD-L1 expression (Figure 4G–K; Supplementary Figure S1). These data suggest that cisplatin-induced PD-L1 in BC occurs mostly through ERK1/2 signal activation.

### Cisplatin induces PD-L1 expression in BC-derived cell lines via AP-1 (c-Jun) transcriptional activation

**Figure 5:**
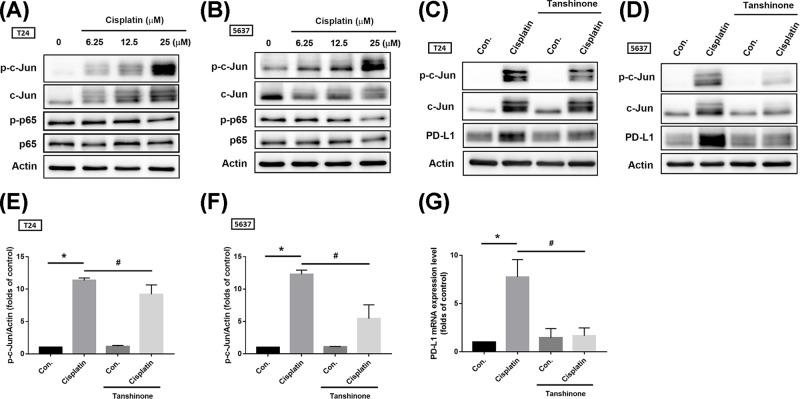
Cisplatin-promoted PD-L1 expression is mediated by the AP-1 (c-Jun) transcription factor (**A,B**) T24 and 5637 BC-derived cell lines were treated with different concentrations of cisplatin for 24 h (6.25, 12.5, or 25 μM), total protein was extracted and phosphorylation of c-Jun and p65 were detected by Western blot. Total p65 and c-Jun was used as the internal control. (**C,D**) T24 and 5637 BC-derived cell lines were pretreated with 50 μM of an AP-1 inhibitor (tanshinone) for 30 min then with cisplatin (25 μM) for 24 h. Total protein was extracted and then c-Jun activation and expression levels of PD-L1 were detected by Western blot. β-Actin was used as the internal control. (**E,F**) The relative band intensities of proteins presented in (C,D) were quantified by densitometric scanning and are presented as the fold change of the control group. (**G**) T24 BC-derived cell lines were treated as described in (C) and total mRNA was extracted from cells; expression levels of PD-L1 were detected by qPCR. Results are expressed as the mean ± S.D of triplicate samples. *P<0.05 compared with the control group and #*P*<0.05 compared with the cisplatin-treated group.

**Figure 6:**
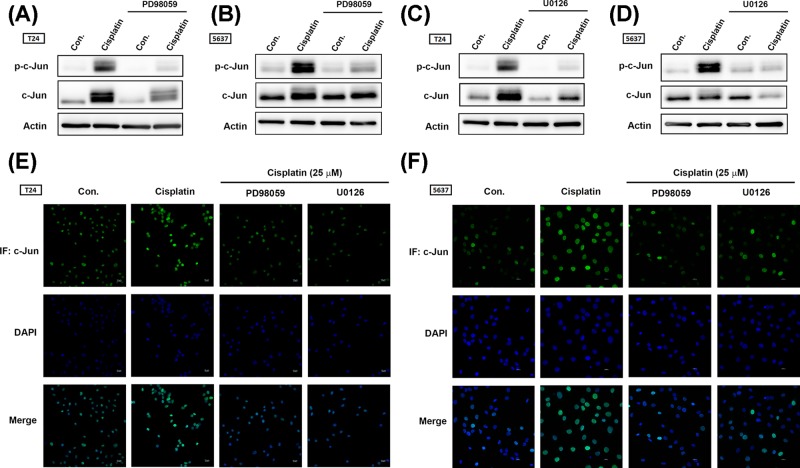
The ERK1/2/AP-1 (c-Jun) signaling cascade contributes to PD-L1 expression in BC-derived cell lines after cisplastin treatment (**A–D**) T24 and 5637 BC-derived cell lines were initially treated with different ERK1/2 pathway inhibitors (PD98059, 10 μM; U0126, 10 μM) for 30 min then with cisplatin (25 μM) for 24 h. Total protein was extracted, then c-Jun activation was detected by Western blot. β-Actin was used as the internal control. (**E,F**) T24 and 5637 BC-derived cell lines were treated as described in Figure 5C,D, then subjected to immunofluorescence by anti-c-Jun antibody staining. Nuclei were counterstained with DAPI. Representative microscopy images are shown. Results are expressed as the mean ± S.D of triplicate samples. *P<0.05 compared with the control group and #*P*<0.05 compared with the cisplatin-treated group.

The AP-1 transcription factor is a dimeric transcription factor that is composed of c-Jun, c-Fos, activating transcription factor (ATF). The main AP-1 proteins in mammalian cells are c-Jun and c-Fos. When the cellular counterparts of the viral oncoproteins were discovered, the up-regulation of AP-1 proteins by overexpression or by oncogenic RAS was found to correlate with a positive effect on cell transformation [13]. MAPK family members, including ERK1/2, JNK, and p38, contribute to activation of AP-1 transcription factor [14]. A previous report has described how a BRAF inhibitor up-regulates PD-L1 in melanoma cells via c-Jun activation [15]. Furthermore, chemotherapeutic drug promotes PD-L1 expression in ovarian cancer through NF-κB activation [16]. We therefore investigated c-Jun and p65 phosphorylation after cisplatin treatment and found that cisplatin significantly induced c-Jun but not p65 phosphorylation in BC-derived cell lines (Figure 5A,B). Pretreatment with the AP-1 inhibitor tanshinone reduced c-Jun phosphorylation and PD-L1 expression (Figure 5C–G). To confirm whether AP-1 activation is regulated by the ERK1/2 signaling pathway, we used ERK1/2 pathway inhibitors. Pretreatment of cells with ERK1/2 pathway inhibitors (PD98059 and U0126) reduced cisplatin-induced c-Jun phosphorylation and nuclear translocation (Figure 6). These results indicate that cisplatin-promoted PD-L1 expression is mediated through the ERK1/2/AP-1 signaling pathway.

## Discussion

The anti-PD-L1 antibody MPDL3280A, a systemic cancer immunotherapy, has proven efficacy in the treatment of metastatic BC; tumors expressing high levels of PD-L1 had particularly high response rates [17]. Here, we found that cisplatin increased PD-L1 expression in BC-derived cell lines. Our finding reveals the mechanism of drug resistance that is regulated by PD-L1 expression after chemotherapy fails in BC. This work provides novel insights for the development of anti-PD-L1 antibodies that can potentially prevent the recurrence of BC after chemotherapy.

Numerous reports have suggested that intrinsic cellular changes are associated with carcinogenesis-induced PD-L1 expression. T-cell lymphoma cells carrying the oncogenic nucleophosmin/anaplastic lymphoma kinase (NPM/ALK), which is involved in malignant transformation, induce high levels of PD-L1 expression via STAT3 and ERK activation [18,19]. Molecular resistance to BRAFi, as exemplified by increased MAPK signaling, prompts PD-L1 expression by enhancing the activity of c-Jun and its cofactor, STAT3 [15]. PTEN negatively regulates the phosphatidylinositol 3-kinase (PI3K)/Akt pathway, alternations of which are also evident in squamous cell carcinoma, together with a reduction in/loss of PTEN. In human glioma, loss of PTEN correlates with enhanced PD-L1 expression [20]. Here, we investigated how candidate signal transduction mediates PD-L1 expression after cisplatin treatment. We found that the ERK1/2 signaling pathway plays a major role in PD-L1 expression after cisplatin treatment. Akt activation was not required for PD-L1 expression. Our result suggests cell type-specific responses in BC-derived cell lines.

AP-1 protein is a well-established pro-oncogenic transcription factor. The activation of AP-1 is rapidly induced by growth factors, cytokines and oncoproteins, which are implicated in the proliferation, survival, differentiation, and transformation of cells [21]. A mouse containing a mutation of this AP-1 site had less PD-1 expression on tumor-infiltrating T cells and demonstrated increased anti-tumor immunity [22]. Another study indicates that AP-1 activation in melanoma cells with BRAFi resistance is closely related to the levels of PD-L1. Moreover, knockdown of c-Jun is necessary and sufficient to suppress the expression of PD-L1 in melanoma cells that are either sensitive or resistant to BRAF inhibition [15]. As with previous evidence, we found that cisplatin contributed to c-Jun activation and thus promoted PD-L1 expression in BC-derived cell lines. Our finding provides a new opportunity for anti-cancer treatments based on AP-1 inhibition.

The MAPK cell signaling pathways play important roles in the regulation of cell growth, proliferation, and survival. Mutations within these pathways are frequently implicated in the pathogenesis of solid tumors [23]. Agents targeting the MAPK pathway that have been used in various combination treatment strategies (vertical inhibition or horizontal inhibition) have significantly benefited patients with different types of tumors [24]. Immunotherapy with immune checkpoint inhibitors that target cytotoxic T-lymphocyte-associated protein-4 (CTLA-4) or PD-1 is another therapeutic approach that has been successfully applied to the treatment of solid tumors [25]. Thus, there is considerable interest in combining immunotherapy with targeted therapy [26,27]. The combination of targeted therapy plus immune checkpoint immunotherapy is currently being evaluated in different cancers including melanoma, non-small cell lung cancer and renal cell carcinoma [28]. Our findings highlight the pivotal role played by the MAPK pathway in cisplatin-promoted PD-L1 expression, especially ERK1/2 activation.

In the present study, we demonstrate that cisplatin promotes PD-L1 expression through the ERK1/2 signaling pathway. This work provides evidence in support of the development of combination therapy using a conventional chemotherapy drug and a PD-L1 antibody for the treatment of BC.

## Supporting information

**Supplementary Figure S1:** The efficacy tests of p38 and JNK inhibitors.

## Abbreviations

AP-1: activator protein-1
BC: bladder cancer
BCG: Bacillus Calmette-Guérin
CTLA-4: cytotoxic T-lymphocyte-associated protein-4
MAPK: mitogen-activated protein kinase
MEK: mitogen-activated protein kinase kinase
PD-1: programmed cell death 1
PD-L1: programmed cell death ligand-1
