# Correction: Cisplatin contributes to programmed death-ligand 1 expression in bladder cancer through ERK1/2-AP-1 signaling pathway

**DOI:** 10.1042/BSR-20190362_COR

**Published:** 2020-08-07

**Keywords:** Bladder cancer, cisplatin, ERK, PD-L1

The authors of the original article “Cisplatin contributes to programmed death-ligand 1 expression in bladder cancer through ERK1/2-AP-1 signaling pathway” (*Biosci Rep* (2019) **39**(9), DOI: 10.1042/BSR20190362) would like to correct errors in their figures. The Western blot images for Figure 6A (p-c-Jun) and 6C (p-c-Jun), as well as Figure 3A (p-mTOR; the 3rd blot from top) and Figure 3B (the 2nd blot from top; Akt) were mistakenly repeated.

**Figure 3:**
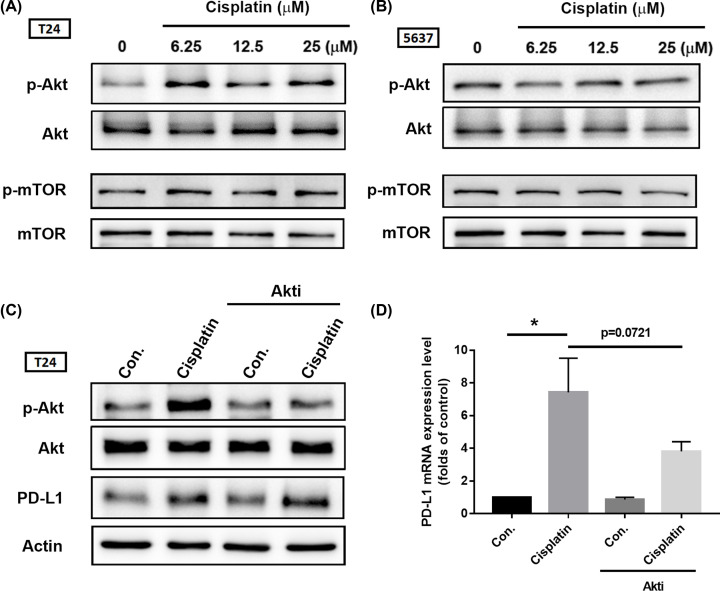
The Akt/mTOR signal pathway is not required for PD-L1 expression after cisplatin treatment (**A,B**) T24 and 5637 BC-derived cell lines were treated with different concentrations of cisplatin for 24 h (6.25, 12.5, or 25 μM, respectively), total protein was extracted and phosphorylation of Akt and mTOR was detected by Western blot. Total Akt and mTOR were used as the internal controls. (**C,D**) T24 and 5637 BC-derived cell lines were initially treated with 3 μM of Akti for 30 min, then with cisplatin (25 μM) for 24 h. Total protein was extracted and subjected to Western blot and qPCR assessments of Akt activation and levels of PD-L1. β-Actin was used as the internal control. Results are expressed as the mean ± S.D of triplicate samples. *P<0.05 compared with the control group.

In their experiment, all results were repeated in more than three independent runs. Due to lack of experience with handling a substantial quantity of Western blot data, mistakes had occurred during the arrangement of the final Figures 3 and 6.

**Figure 6:**
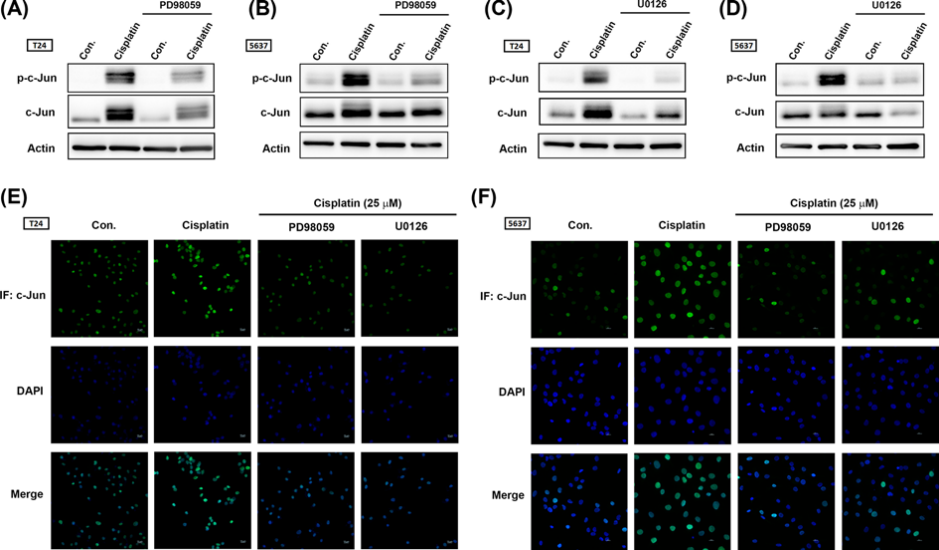
The ERK1/2/AP-1 (c-Jun) signaling cascade contributes to PD-L1 expression in BC-derived cell lines after cisplastin treatment (**A-D**) T24 and 5637 BC-derived cell lines were initially treated with different ERK1/2 pathway inhibitors (PD98059, 10 μM; U0126, 10 μM) for 30 min then with cisplatin (25 μM) for 24 h. Total protein was extracted, then c-Jun activation was detected by Western blot. β-Actin was used as the internal control. (**E,F**) T24 and 5637 BC-derived cell lines were treated as described in Figure 5C,D, then subjected to immunofluorescence by anti-c-Jun antibody staining. Nuclei were counterstained with DAPI. Representative microscopy images are shown. Results are expressed as the mean ± S.D of triplicate samples. *P<0.05 compared with the control group and #*P*<0.05 compared with the cisplatin-treated group.

The corrected Figures 3 and 6 are presented in this article. The authors sincerely apologise for this mistake and wish to amend their error. The results and conclusion of their study remain the same.

