# Supplementary material for: Cisplatin contributes to programmed death-ligand 1 expression in bladder cancer through ERK1/2-AP-1 signaling pathway
Source: Biosci Rep. 2019 Sep 6;39(9):BSR20190362. doi: 10.1042/BSR20190362 (PMC6783655; doi:10.1042/BSR20190362)
Supplement: Supplementary file 1 [file bsr20190362_Supp1.pdf]

Supplementary information

Figure S1

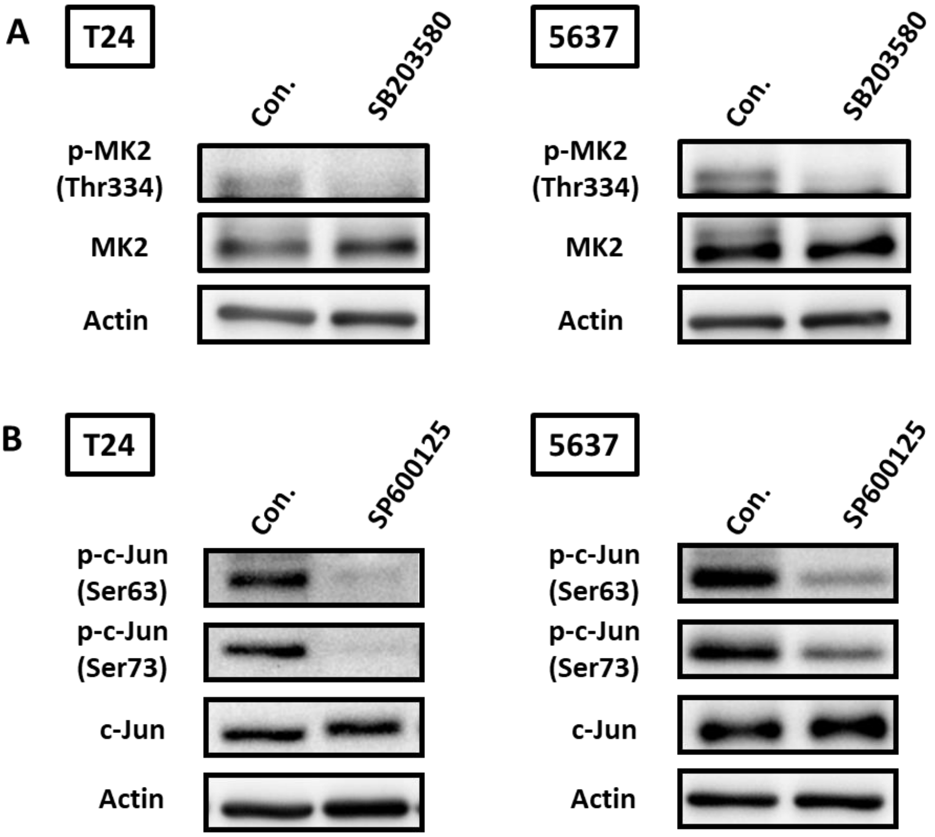

Figure S1. The efficacy tests of p38 and JNK inhibitors. (A and B) The BC-derived cell lines were incubated with SB203580 (10 μM) or SP600125 (10 μM) for 24 h. The cell lysates were collected and phosphorylation of MK2 and c-Jun were conducted by Western blot respectively.
